# Current Strategies to Enhance Anti-Tumour Immunity

**DOI:** 10.3390/biomedicines6020037

**Published:** 2018-03-23

**Authors:** Katherine W. Cook, Lindy G. Durrant, Victoria A. Brentville

**Affiliations:** 1 Scancell Limited, Academic Department of Clinical Oncology, University of Nottingham, City Hospital Campus, Nottinghamshire NG5 1PB, UK; katherinecook@scancell.co.uk (K.W.C.); VictoriaBrentville@scancell.co.uk (V.A.B.); 2 Academic Department of Clinical Oncology, Division of Cancer and Stem Cells, School of Medicine, University of Nottingham, City Hospital Campus, Nottinghamshire NG5 1PB, UK

**Keywords:** vaccination, oncolytic viruses, adoptive cell transfer, checkpoint inhibition, immune modulators, immune surveillance

## Abstract

The interaction of the immune system with cancer is complex, but new approaches are resulting in exciting therapeutic benefits. In order to enhance the immune response to cancer, immune therapies seek to either induce high avidity immune responses to tumour specific antigens or to convert the tumour to a more pro-inflammatory microenvironment. Strategies, including vaccination, oncolytic viruses, and adoptive cell transfer all seek to induce anti-tumour immunity. To overcome the suppressive tumour microenvironment checkpoint inhibitors and modulators of regulatory cell populations have been investigated. This review summarizes the recent advances in immune therapies and discusses the importance of combination therapies in the treatment of cancers.

## 1. Introduction

The concept that the immune system can recognize and eliminate cancerous cells is referred to as immune surveillance [1,2]. Immunological differences between cancer and self can be detected naturally and lead to the elimination of cancerous cells in many individuals. This theory suggests that the immune system is naturally capable of detecting and eliminating cancer cells. Studies in immunocompetent mice show the rejection of tumours and the protection gained can be adoptively transferred to mice via transfer of the T-cells [3]. In mice, knockout models showed that interferon gamma (IFNγ) and lymphocytes are important in reducing the incidence of carcinogen-induced sarcoma and spontaneous epithelial carcinomas [4]. Patients with spontaneously regressing melanoma showed signs of tumour-specific clonal T-cell expansion providing evidence of immune surveillance [5]. Recent work has demonstrated that the mutations accrued by tumours during the transformation process can generate neo-antigens that can be efficiently targeted by T-cells [6]. This work all highlights the fact that the immune system is capable of distinguishing between tumour and self, but cancer still develops in many immunologically healthy individuals. This is due to the high mutation frequency of tumours, which can lead to immune equilibrium where the tumour avoids the original immune response but the immune response can adapt and still attack the tumour. Eventually the tumour escapes in a process termed “immune editing” and grows despite the immune response [7].

One of the major factors to consider when discussing tumour immunogenicity is the heterogeneity within and between cancers. Not all cancers have the same mutational load [8] and this has recently been shown to play a role in immune infiltrate and the response to immunotherapy [9,10]. Classification of tumours according to the immune cell infiltration (immunoscore) has begun to help immunologists to predict how they might respond to different immune based treatments [11]. Tumours can be classified as “hot” where they have a high number of tumour infiltrating immune cells, or “cold” where they have limited tumour infiltration. “Excluded” tumours exhibit immune cells found only in the periphery of the tumour and in the most extreme case “cold” tumours which are devoid of immune cell infiltrate are referred to as “immune desert” [12]. The type of immune infiltrate that is found in tumours also has a profound effect on the clinical outcome and response to therapies. Tumours with greater T-cell infiltrate do have better prognosis. For example, analysis of pre-treatment melanomas showed that patients with higher baseline immune gene profiles in tumour biopsies were more likely to respond to checkpoint inhibitor treatment [13].

Immune therapies seek to recruit immune cells into the tumour microenvironment and enhance anti-tumour immunity. There are two major reasons that could prevent this from occurring. Firstly, tumours do not present the correct environmental conditions to prime an effective immune response. In the absence of co-stimulation induced by signals, such as Toll-like receptors (TLRs) or damage-associated molecular pattern molecules (DAMPS) to stimulate danger or sufficient presentation of tumour specific epitopes to prime responses, high avidity T-cells that are capable of tumour lysis are not generated [14,15]. The stimulation of low avidity T-cells that cannot kill tumour cells is believed to be the reason for the failure of many early tumour vaccines. Strategies to address the lack of T-cell priming and induce tumour-specific immune responses include vaccines and adoptive cell transfer (ACT). These strategies require the identification of appropriate tumour antigens and adjuvants to provide co-stimulation that ensures robust responses are induced. The second barrier is the suppressive tumour environment that prevents the infiltration or function of immune cells [16]. If tumour specific T-cell responses are present but the T-cells are not effective, then in situ cancer cells will not be eliminated [17]. Strategies such as checkpoint blockade and immune modulators seek to overcome this issue by reversing immune suppression and promoting a pro-inflammatory environment [18].

This review will discuss current strategies to induce better anti-tumour immune responses and those being employed to overcome the immune suppressive tumour environment. Finally, the promise of combination therapies will be discussed.

## 2. Selection of Target Antigens That Provide Increased Tumour Antigenicity

Efficient anti-cancer immunotherapy relies on effective targeting of antigens that can be recognized by high avidity T-cells. Many antigens expressed on tumours are also expressed on normal tissues and the T-cells recognizing them are subject to thymic tolerance. This leaves a repertoire of low avidity T-cells that can be stimulated by high doses of immunogen, but that can never see sufficient target on the tumour cells to kill them. The goal is to find antigens that are not thymically expressed and are found in high abundance on tumour cells but not healthy tissues.

### 2.1. Mutated Antigens and Neo-Antigens

Tumours accumulate mutations that drive growth and metastases. These mutations represent unique epitopes that avoid thymic selection. They are termed “neo-epitopes” and are specific to individual tumours and are not found on normal tissues [19]. Lennerz et al. identified in a mixed lymphocyte-tumour cell cultures from one patient with long-term survival of melanoma responses to eight antigens, five of which were neo-antigens [20]. This is an early study that showed that neo-antigens are associated with responses in long-term survivors. This has led to researchers to develop personalized vaccines against identified neo-epitopes. Not all of the mutations stimulate a T-cell response but there is a correlation between the frequency of mutations and the likelihood of presenting a T-cell epitope. Indeed, patients with tumours that have higher mutation rate often show better responses to checkpoint blockade therapies suggesting endogenous neo-epitope responses are uncovered by the checkpoint blockade [9]. As most mutations do not stimulate an immune response, selection of the most appropriate epitope to target can be difficult. However, significant progress is being made in the appropriate selection of candidate epitopes [21].

In human melanoma mass spectrometry has been used to identify neo-epitopes directly from primary tumour leading to the identification of a number of potential targets [22]. Therapies targeting these neo-epitopes are being translated into the clinic and showing the efficient induction of specific immune responses [23,24,25]. Several groups have treated patients with vaccines targeting multiple neo-epitopes. Sahin et al. have shown that multiple neo-epitope specific responses can be generated in patients after treatment with intranodal delivery of a RNA polyepitope vaccine [24]. They demonstrated a decrease in metastatic events and sustained progression free survival. Ott et al. also treated six melanoma patients with a peptide polyepitope vaccine combined with the adjuvant Hiltonol (a stabilisation of poly IC with poly-l-lysine double-stranded RNA). They saw efficient neo-epitope specific T-cell responses with reduced recurrence rate [25]. In addition, patients with recurrent disease post vaccination exhibited a complete regression after subsequent anti-Programmed cell death (PD-1) therapy, which was associated with the expansion of neo-epitope specific T-cell responses. Interestingly the neo-epitopes identified in these studies were recognized by both CD8 and CD4 T-cells suggesting an important role for CD4 T-cell responses in addition to CD8 responses in humans. This confirmed previous data obtained in mouse models by Kreiter et al. [26].

One disadvantage of targeting neo-antigens is they are expensive as they are patient specific and there can be huge variability both within a tumour sample and between tumours in the same patient, this can lead to outgrowth of tumours that no longer express the mutation [27]. To overcome “driver” mutations that are key in the tumorigenesis process (e.g., BRAFV600E), or other common mutations, they can be specifically targeted; however, these are much rarer and do not always stimulate T-cell responses.

### 2.2. Post-Translational Modifications

Another form of neo-epitope not previously explored in much detail is the post-translationally modified epitopes that are associated with cancer. Like neo-epitopes these post-translationally modified epitopes are likely to have escaped thymic deletion and are expressed by a wide range of cancers.

Cancer cells often exhibit changes in phosphorylation patterns and this has led to interest in using phosphorylated antigens as targets for vaccines [28,29,30]. One study has recently demonstrated that mass spectrometry analysis of tumour biopsies can identify phosphopeptides as a way of identifying phosphorylated targets [31]. This makes the phosphoproteomics of biopsies a viable option to identify potential epitopes allowing another aspect of personalised treatment to be developed. CD8 and CD4 T-cells can be detected against tumour specific phosphopeptides but these have yet to prove efficacy in a clinical setting due to the instability of the phosphorylated peptides [28,30].

Our research has recently focussed on epitopes, which are citrullinated in tumour cells. Citrullination is a post translation modification where arginine residues are converted into citrulline by the peptidylarginine deaminase (PAD) enzymes [32,33]. PAD enzymes function in the presence of high calcium that is present during stress induced autophagy. Physiologically citrullination can serve several functions, including the regulation of genes and the immune response. However, during stress induced autophagy it has been shown that citrullinated peptides can be presented on major histocompatibility complex (MHC) class II molecules for recognition by CD4 T-cells [34]. Tumour cells are often subject to a “stressed” environment specifically through hypoxia and nutrient starvation common in rapidly growing tumours. Their survival depends upon autophagic flux to provide energy and nutrients for growth. We have shown that potent cytotoxic CD4 T-cells can be induced to citrullinated peptides and these provide efficient tumour therapy in aggressive murine tumour models, with no associated toxicity [35,36]. Most tumours do not express MHC-II, but CD4 T-cells are initially activated by tumour infiltrating antigen presenting cells (APCs) that constitutively undergo autophagy and express MHC II. Activated CD4 T-cells release pro-inflammatory cytokines, such as IFNγ, which in turn, upregulate MHC-II expression on tumours to allow for direct recognition by CD4 T-cells. Indeed, the tumour immunity that was observed was reliant upon the CD4 T-cells, but independent of CD8 T-cells [35]. We have demonstrated the targeting of citrullinated vimentin, and enolase that are expressed by a wide range of cancers. However, a number of other cytoskeletal, regulatory, chaperone, and glycolytic proteins have been shown to be citrullinated during cellular stress and these provide further potential targets for tumour immunity [37,38]. Targeting of stress induced post-translational modifications has yet been tested in clinical studies but is a potential area of expansion for target selection. We hypothesis that targeting of stress induced post-translational modifications has the potential to both generate de-novo immune responses and boost pre-existing responses and be applicable to cancers where low T-cell infiltrate occurs. Other post-translational modification being examined in pre-clinical studies include DNA methylation and epigenetic modifications [39], and glycolysis [40].

### 2.3. Tumour Associated Antigens and Cancer Testis Antigens

High avidity T-cells that are specific for self-antigens are routinely deleted in the thymus during development, leaving a low avidity repertoire. Therefore, antigens that show limited normal expression are likely to act as better targets since they may not have been subject to the same degree of tolerance. The detection of T-cells specific to self-antigens in regressing cancer patients suggests that thymic tolerance is not always complete. Regressing cancer patients made responses predominantly to antigens with restricted expression in normal tissue, such as the differentiation antigen TRP-2 and the cancer testis antigen NY-ESO-1 [41,42]. Tumour associated antigens (TAA), including NY-ESO-1 [41,43] and the melanoma antigen MAGE-1 [44], make good targets for the immune response suggesting that they have subverted immune tolerance.

One problem with targeting tumour specific antigens is that mutation in the tumour may result in antigen loss variants, which can evade the action of the immune responses generated. A way to overcome this is to target multiple TAA in a single a vaccination. DNA vaccines have successfully combined a number of antigens. SCIB1 is a DNA vaccine that contains epitopes for glycoprotein 100 (gp100) and TRP-2 and has shown promising early results in patients [45]. SCIB1 induced dose-dependent T-cell responses in 88% of patients with no serious adverse effects or dose limiting toxicities. The intensity of the T-cell responses was significantly higher in patients receiving 4 mg doses without tumour when compared to those with tumour (*p* < 0.01). In contrast, patients with a tumour showed a significantly higher response to the 8mg dose than the 4 mg dose (*p* < 0.03), but there was no significant difference in the patients without a tumour. This suggests that a higher dose of vaccine is required for patients with tumours. One of 15 patients with measurable disease showed an objective tumour response and 7/15 showed stable disease. 5/20 fully-resected patients have experienced disease recurrence but all remained alive at the cut-off date with a median observation time of 37 months. A positive clinical outcome was associated with MHC-I and MHC-II expression on tumours prior to therapy (*p* = 0.027). Another approach uses peptides to induce response to TAA identified in the tumour by genome-wide cDNA microarrays [46]. Vaccination with a mixture of three different cancer testes antigens induced TAA-specific T-cells and anti-tumour activity in the head and neck cancer patients [47,48].

### 2.4. Viral Antigens and Infectious Agents

A number of cancers are associated with viral infection such as Epstein Barr Virus in Burkitt’s lymphoma, Human Papilloma Virus in cervical cancer, and Hepatitis B and C viruses in hepatocellular carcinoma. In addition to virus associated cancers, gastric cancers are known to be associated with *Helicobacter pylori* infection [49]. These cancers that are driven by infectious agents often express certain antigens that have not been subject to immune tolerance and can be efficiently targeted by the immune system. Indeed, immune responses can be efficiently induced to these infectious agents that protect against cancer development if administered before exposure to the infectious agent or during pre-malignant disease. This is exemplified in successful vaccines against Hepatitis B virus and Human Papilloma Virus [50]. More limited success has been had in therapeutic approaches targeting viral antigens [51,52,53]. This is in part due to the ability of these infectious agents to modulate and subvert the host immune response, and also to peripheral tolerance mechanisms that operate during persistent infections [54,55].

## 3. Mechanisms to Enhance Tumour-Specific Immune Responses

### 3.1. Vaccination

Once an appropriate antigen has been selected, then it is important to consider how best to present it to the immune system. Stimulation of T-cells requires the processing and presentation of antigen by professional APCs, such as dendritic cells (DCs), along with appropriate activating costimulatory signals. Activating costimulatory signals include those provided by TLR ligands [56]. Preclinical studies examining linkage of the peptide vaccine directly to TLR ligands are beginning to show promise. These are thought to more efficiently target both epitope and TLR to DCs, leading to increased DC maturation and the expression of costimulatory molecules, secretion of cytokines and chemokines, and the formation of an antigen depot within DCs to allow for prolonged presentation of the peptide [57,58]. In addition to direct linkage, studies have investigated the use of amphiphilic peptides combined with TLR ligands that assemble into nanostructures and are showing promise in preclinical studies [59,60]. It is also important to consider the dose of antigen that is provided by the vaccine. A low dose can be enough to select for highest affinity T-cell receptor (TCR) and thus high avidity CD8 T-cells [61], but it may not be sufficient to stimulate CD4 T-cells whose epitope target demonstrates lower affinity MHC-II binding.

Peptide vaccines encoding tumour epitopes have shown promise in animal models in early studies, stimulating specific T-cell responses and tumour therapy in mice. Translation of these peptide vaccines into the clinic has been less successful with responses being short lived and minimal clinical efficacy. Early vaccines concentrated on the stimulation of CD8 T-cell responses with short (<15 amino acids) peptides. However, more recent studies focus on the use of longer peptide sequences that can stimulate both CD4 and CD8 T-cell responses to avoid problems with tolerisation previously seen with shorter peptide sequences [62]. Longer peptide sequences are beginning to show promising results in clinical studies [63,64]. Peptides encoding neo-epitopes are also beginning to show some potential with the detection of robust immune responses and evidence of improved overall survival [65,66]. A study by Ott et al. (2017) demonstrated enhanced neo-epitope specific responses after vaccination, with 20 amino acid long peptides being mixed with the TLR3 ligand Hiltonol [25].

Synthetic peptides have also been used as part of DC based vaccines. Many studies have been performed where DCs cultured in vitro have been pulsed with peptides, proteins, or tumour lysates. These have shown stimulation of efficient immune responses in preclinical studies [67]. Despite stimulating immune responses, DC vaccines have shown limited efficacy in the clinic. Sipuleucel-T (Provenge^®^), the only approved therapeutic autologous cell based vaccine to date, has shown a modest survival benefit of three months, but the cost and time of production have severely limited its use [68]. This is the major limiting factor of most DC and autologous cell-based vaccines. In addition, a DC vaccine incorporating neo-epitope peptides showed a promising expansion of both existing and de novo neo-epitope specific responses [23]. The use of immature DCs can impact their immunogenicity and lead to tolerance induction therefore the activation state of DCs used needs to be closely monitored. The extensive culture methods to manufacture DC based vaccines may also impact on their immunogenicity in vivo. Recent work focusing on the isolation of DC subsets ex vivo with minimal in vitro manipulation has shown promising results [69].

Nucleic acid vaccines have increased in popularity within the last 20 years. The appeal of nucleic acid vaccines is they are relatively quick, easy and cheap to make and they are one of the only recombinant vectors that do not stimulate immunity to the vector itself. RNA and plasmid DNA both possess intrinsic adjuvant activities, which aid both recruitment of immune cells to the site of immunisation and the activation of the immune cells. A disadvantage of DNA vaccines is the inefficient uptake into APCs of naked DNA. This uptake can however be enhanced through the use of electroporation or nanoparticle technologies [70,71,72]. Our work with DNA vaccination utilised a novel approach where the DNA encodes for a humanised IgG1 antibody in which the CDR regions are replaced with T-cell epitopes. The Fc region of the IgG1 antibody targets the high affinity Fc receptor CD64 that is expressed on activated APCs, but not immature APCs [61,73]. The inclusion of the T-cell epitopes within the CDR regions leads to improper antibody folding and results in low levels of protein produced. This in turn stimulates higher avidity CD8 responses when compared to peptide or DC based vaccination [61]. The use of electroporation to successfully deliver a DNA vaccine encoding epitopes in the CDR regions of a human IgG1 antibody (SCIB1) in melanoma patients was well tolerated and showed minimal toxicity. SCIB1 vaccine targeting melanoma differentiation antigens gp100 and TRP-2 induced dose dependent T-cell responses in 88% of patients and is evidence of clinical responses [45]. The provision of T-cell help has been considered for some time a vital component for efficient induction of immune responses. A DNA vaccine encoding a tumour specific CD8 epitope fused to a universal CD4 epitope showed enhanced immune responses. Electroporation was also used to enhance DNA uptake in these studies. Detectable tumour antigen specific responses were seen in prostate cancer and colorectal cancer patients, which was associated with some signs of clinical efficacy [74,75].

RNA has also been investigated for vaccine delivery. Since RNA is inherently unstable several groups have examined stabilised RNA formulations and these can be shown to stimulate immune responses although anti-tumour responses are variable [76]. A similar approach targeting tyrosinase and the NY-ESO-1 cancer testis antigen was undertaken using RNA complexed with liposomes. Intranodal delivery of this vaccine resulted in stimulation of NY-ESO-1 specific T-cell responses [24]. The same study also examined the responses to neo-epitopes using the RNA based vaccination strategy and showed successful immune responses and promising clinical outcomes.

### 3.2. Oncolytic Viruses

Oncolytic viruses are able to selectively replicate in cancer cells and kill without harming normal tissue. Oncolytic viruses can be either genetically engineered or naturally occurring viruses that have the ability to induce cancer cell death and tumour-specific immunity [77]. Cancer cells have impairments that mean they are more susceptible to viral infections than normal cells. However, genetic engineering has been utilised in many oncolytic viruses to further impair the ability of the virus to infect and replicate in normal tissues.

The oncolytic virus Talimogene laherparepvec (T-vec), which is a second-generation oncolytic herpes simplex virus type 1 (HSV-1) with deletions in two viral genes and the addition of Granulocyte-macrophage colony-stimulating factor (GM-CSF) is now licensed for use against melanoma. T-vec specifically infects and replicates in human tumour cells and is associated with a significant reduction in tumour growth in a number of in vivo models [78]. The addition of either the human or mouse GM-CSF gene means that T-vec can attract and mature APCs, leading to increased presentation of tumour antigens in the tumour microenvironment [79]. GM-CSF is used as an adjuvant in some cancer vaccines and it is hoped it will have a similar effect when produced by the oncolytic virus. In a phase III trial, patients with stage III and IV melanoma, intralesional injection of T-vec had a good responses rate, which was higher than that observed in the arm of the trial given GM-CSF only [80].

Other oncolytic viruses in development include the vaccinia viruses pexastimogene devacirepvec (JX-594) and TG6002 [81] which have both been shown to be effective at lysing melanoma cells and inducing inflammation [82]. For JX-594 a mutation in the *TK* gene makes the virus cancer cell selective and human GM-CSF has been added to increase immunogenicity [83,84]. A randomised control trial has been carried out using JX-594 in patients with hepatocellular carcinoma. This study suggested a dose-dependent effect on survival [85]. The reovirus Reolysin is a naturally occurring oncolytic virus that has been targeted to patients with head and neck cancer, among a number of other indications [86]. Patients with advanced solid tumours were treated with the combination of carboplatin and paclitaxel chemotherapy and Reolysin in a phase I/II trial and nine out of thirty-one patients had some form of response, nine had stable disease, and eight showed disease progression [87]. The safety of oncolytic viruses will need continued scrutiny to confirm that transmission is not possible and that the viruses are not able to mutate or recombine to produce pathogenic forms. Apart for the safety aspect the major limitation of oncolytic viruses will be whether high viral titers can be delivered to the tumour [88]. Most successful studies have relied on intralesional delivered virus, which is not a viable option for most solid tumours. Systemic delivery is also hampered by immune responses to the virus. Oncolytic viruses can be subjected to the action of circulating neutralising antibodies. In one clinical trial, i.v. administration of oncolytic measles virus (MV-NIS) in patients with multiple myeloma was shown to be less effective due to neutralising antibodies [89]. In murine models increased tumour growth was associated with increased serum anti-reovirus antibody titers [90].

### 3.3. Adoptive Cell Transfer and Chimeric Antigen Receptor T-Cells

Another strategy that has been developed uses adoptive transfer of ex vivo expanded immune cells that are capable of responding to tumour or to a specific tumour antigen. ACT requires the isolation of lymphocytes from patients, expansion in vitro and injection back into the patient. Classically these lymphocytes are isolated from the patient’s tumour and this technique has shown some promise in the clinic [91,92]. Studies have shown that many tumour infiltrating lymphocytes (TILs) recognize neo-antigens [93], and more recently the autologous transfer of T-cells specific to these mutated antigens provided durable clinical responses [94]. However, ACT is not always successful and despite showing persistence of transferred T-cells, studies in melanoma isolating CD8+ TILs specific for TAA gp100 and MART1 showed little clinical effect [92]. It is possible that this is linked to suppressive effects of the tumour environment or perhaps the phenotype of the cells after manipulation of the cells in vitro prior to adoptive transfer.

Another method for ACT is to avoid the isolation of TILs from patient tumours and to genetically engineer patient T-cells to express a TCR recognizing a specific tumour antigen. Limitations for this approach lie with the identification of an appropriate TCR sequence that has sufficient affinity to target the peptide MHC complex within tumours, whilst minimising any off-target toxicity. This is highlighted in early studies targeting the TAA MART1 [95,96]. However, the modification of TCRs to high affinity can result in associated “off target” toxicity that is thought to be caused by high levels of inflammatory cytokines or molecular mimicry of unrelated epitopes expressed by normal tissues [97,98]. The targeting of a single epitope in combination with a single MHC allele will increase the likelihood of immune editing, resulting in antigen/epitope or MHC allele loss variants. Therefore, the design of genetically engineered T-cells should include the consideration of multiple MHC allele and antigen targets that will minimise the off-target toxicity but maximise the T-cell function. Progress to this extent has been achieved with therapies targeting the cancer testis antigen NY-ESO-1 where patients showed clinical responses without any associated toxicity [99].

In addition, others have investigated the use of genetically engineered T-cells expressing a chimeric antigen receptor (CAR) specific for a tumour antigen. CAR T-cells are designed to circumvent issues with the recognition of peptides from tumour antigens in complex with MHC molecules. Instead they use an antibody fragment to target a highly expressed cell surface antigen. These cells are modified in a number of ways to allow efficient T-cell activation. They are engineered to express an extracellular single-chain antibody fragment (scFv), which is responsible for antigen specificity and can be altered depending on the tumour target. Cells also have an engineered transmembrane domain, which often includes costimulatory factors, such as CD28 and 4-1BB (CD137), bypassing issues such as downregulation of MHC and the need for costimulatory molecules. Subsequent generation of CAR T-cells have utilised different costimulatory molecules. For example, 4-1BB has been shown to be more effective than CD28 for the expansion of cytotoxic CD8 cells [100]. This has proved to be a balancing act between inducing sufficient responses to eliminate cancer without causing toxicity [101]. Selecting the optimum avidity may be important in finding this balance [102].

The use of CAR T-cells to treat acute myeloid leukaemia (AML) has shown how effective this technique could prove to be at targeting some forms of cancer. AML is a rare disease with poor prognosis, particularly in older patients. The first CAR T clinical trial in AML targeted Lewis Y antigen using second generation CAR T-cells, among the four patients in this trial all showed some clinical effect and none showed severe toxicity [103]. However, the most successful CAR T-cells target is CD19. In one trial, two children were treated with anti-CD19 CAR T-cells, resulting in remission without any graft-versus-host disease, although one child showed the relapse of CD19-tumour [104]. In another trial, patients with acute lymphoblastic leukaemia (ALL) were treated with low dose CD19-directed CAR T-cells. This study showed a good safety profile and 90% of patients (36/40) showed complete remission [105]. In 2017 the FDA approved the use of the CD19-targeting CAR T-cell therapy tisagenlecleucel (Kymriah, Novartis, Basel, Switzerland) for treatment of patients of 25 years or under with ALL and axicabtagene ciloleucel (Yescarta, Kite Pharma, Los Angeles, CA, USA) for adults with ALL. In a multicentre trial of children and young adults with ALL, tisagenlecleucel therapy was associated with event-free survival in 73% of patients and overall survival in 90% of patients [106]. A phase I clinical trial with axicabtagene ciloleucel showed a responses rate of 82% and a complete responses rate of 54% among 111 adult patients [107]. Recent clinical trials using CAR T-cells have also targeted a number of other AML related antigens, including FLT-3 and CD123, with the aim to reduce off-target effects and increase efficacy [108].

While CAR T-cells have been used to successfully treat haematological malignancies, such as leukaemia, they have been less successful in solid tumours [109]. But, CAR T-cells against many different antigens are being developed. In melanoma, preclinical studies have developed CAR T-cells targeting ganglioside GD2 and incorporating 4-1BB [110]. A case study of a patient with recurrent multifocal glioblastoma who was given multiple infusions of CAR T-cells engineered to target interleukin-13 receptor alpha 2 (IL13Rα2) showed good tolerance and regression of intracranial and spinal tumours [111]. Patient-derived tumour cell lines have been used in mouse models to further optimise the CAR T-cells targeting IL13Rα2 and determine the best delivery system to treat clinical brain tumours [112]. Another strategy to enhance the effectiveness of CAR T-cells is to optimise the type of T-cells used. The effector/memory status of input cells has been shown to be important for determining the expansion and anti-tumour activity of CAR T-cells in vivo [113]. The future for CAR T-cells in the treatment of solid cancers will rely on improving tumour extravasation as well as the overcoming the barriers of the suppressive tumour environment.

## 4. Overcoming T-Cell Regulation and Immune Suppression

A problem for strategies that seek to induce an immune response to tumour is anergy or the exhaustion of T-cells. Lee et al. showed early in disease that melanoma patients had circulating CD8 cells that were specific for TAAs, but that these cells seemed to have been rendered anergic, unlike other responses in the patient, such as responses to Epstein Barr Virus (EBV) [114]. The presence of persistent antigen in the tumour environment will also lead to the constant stimulation of T-cells, thus leading to an exhausted non-functional phenotype. T-cell priming and effector functions are determined by interactions with costimulatory or inhibitory receptors, both of which can be targets of immunotherapy.

### 4.1. Agonists against Co-Stimulatory Receptors

Costimulatory receptors are expressed by T-cells during activation and bind to counter receptors that are expressed by APCs. They provide signals that act synergistically with TCR signals to promote T-cell expansion, survival and effector function. Agonistic antibodies that are directed against costimulatory molecules, such as 4-1BB (CD137), OX40 (CD134), CD27, glucocorticoid-induced tumour necrosis factor receptor (TNFR) family-related gene (GITR), ICOS, and CD28 have been developed for use as cancer therapies. For example, OX40 is expressed on both CD4 and CD8 cells during antigen-specific priming and interactions with its natural ligand OX40L promote cell survival and expansion [115]. In preclinical studies, the injection of OX40 agonists significantly improved survival [116]. Another costimulatory receptor is CD27, which is expressed on many T-cells, including naïve T-cells, and has an anti-tumour effect in preclinical models [117]. A number of clinical trials targeting OX40 or CD27/CD70 are ongoing for indications, including melanoma and ovarian and prostate cancer [118]. 4-1BB is another inducible costimulatory receptor that is being targeted as a cancer therapeutic. 4-1BB is expressed on activated T cells and NK cells and agonists for this molecule have been shown to lead to tumour rejection in preclinical models [119]. Clinical trials that use two antibodies to 4-1BB (urelumab and utomilumab) are ongoing. One of the major drawbacks of this strategy is that these antibodies have been shown to have on-target off-tumour effects due to the expression on non-tumour specific T-cells [119,120]. However, a number of studies have shown that combination of these antibodies with other strategies can be effective and current clinical trials are often for combinations of costimulatory agonists and checkpoint inhibitors.

### 4.2. Checkpoint Blockade

Checkpoint inhibitors work by blocking the inhibitory pathways of the immune response and therefore lead to general activation of immune cells. Checkpoint inhibitors, such as anti-PD-1 and anti-CTLA-4 antibodies, have been shown to have good therapeutic effects in many tumours, leading to significant durable survival, thus a number of these are currently approved for clinical use (Table 1). Cytotoxic-T lymphocytes-associated antigen 4 (CTLA-4) is an inhibitory T-cell receptor that competitively antagonises the costimulatory interaction between CD28 and B7 ligand. CTLA-4 expression is upregulated on T-cells following activation and acts as a regulatory feedback to attenuate and terminate the activated T-cell. Anti-CTLA-4 antibodies, such as Ipilimumab and Tremelimumab, act to block this action and lead to persistent T-cell activation. Anti-CTLA-4 antibodies have also been shown to broaden the immune response as well as deplete tumour resident regulatory T-cells (Tregs) [121,122]. Taking the brakes off the immune responses through inhibition of CTLA-4 signalling would overcome the negative regulation and perhaps the reverse exhaustion of T-cells. Indeed, the blocking of certain checkpoints can restore the functionality of tumour associated T-cells [123]. However, this restoration of function can also be associated with an increased autoimmunity and associated toxicity [124].

PD-1/Programmed cell death ligand 1 (PD-L1) signalling is also being successfully targeted by monoclonal antibodies. PD-1 is upregulated on activated T-cells and when it interacts with its ligand PD-L1 leads to the inhibition of T-cell function [125]. One of the major ways that tumours appear to avoid immune surveillance is the upregulation of PD-L1 on the tumour cell surface. This can occur in response to immune pressure, especially in the presence of IFNγ, in a process known as “acquired resistance” [126], and thus, inhibiting the function of T-cells within the tumour environment. Our current understanding suggests that CTLA-4 limits central lymphatic T-cell activity whereas PD-1 signalling is more important in later stages of the immune response in peripheral tissues [127]. Combinations of these two types of checkpoint inhibitor are also being assessed in the clinic with increased efficacy but also increased toxicity. The anti-CTLA-4 antibody Ipilimumab was first shown to be effective in patients against advanced melanoma [128,129].

The most clinically defined responses seen with checkpoint blockade are against melanoma; however, checkpoint inhibitors have also been licenced for use in lung cancer, Hodgkin’s lymphoma, and head and neck cancer, and are being tested in many other tumour types.

### 4.3. Regulatory T-Cells

Tregs play a role in the suppression of immune responses, the maintenance of immune tolerance, and the prevention of autoimmunity. Tregs are a subtype of CD4 cells, which have an immune suppressive function that allows for immune homeostasis to be maintained. In cancer, infiltration of Tregs into the tumour microenvironment has been associated with poor prognosis and poor responses to immune therapy [130]. In animal models, the removal of Tregs has been shown to improve anti-tumour immune responses although it was also associated with increased autoimmunity [131].

CTLA-4 is highly expressed on Tregs and treatment with anti-CTLA-4 antibody in murine studies has been shown to deplete Tregs by a mechanism that is reliant on tissue resident macrophages [122]. However, it has also been shown that enhanced tumour therapy mediated by the anti-CTLA-4 antibody is dependent upon interaction with both Tregs and T-effectors in order to elicit full tumour protection [132]. Therefore, it is possible that the mechanism by which Ipilimumab functions covers both the depletion of Tregs and the blockade of negative signalling although this has yet to be proven in patients.

### 4.4. Tumour Microenvironment

In addition to Tregs other factors in the tumour microenvironment contribute to the suppression of immune responses. Myeloid derived suppressor cells (MDSCs) originate from a myeloid lineage and develop as a heterogeneous population that is influenced by pro-inflammatory mediators that are produced by both tumour and normal cells. Activated MDSCs possess suppressive activity that promotes immune tolerance and enhances tumour growth [133]. Many strategies have been investigated in preclinical studies to target MDSCs, including targeting their mechanisms of suppression and blocking their differentiation and development. Only a small number have reached clinical studies where they have shown some efficacy in the reduction in MDSC numbers and function and were associated with enhanced immune responses [134,135,136,137].

Indoleamine 2,3-dioxygenase (IDO) is an enzyme that is important in the degradation of tryptophan during cellular metabolism. It plays roles in cancer progression, generation of immune tolerance, causes increased apoptosis in T-cells through the depletion of tryptophan, and promotes differentiation of CD4 T-cells into Tregs [138]. IDO can also be induced in response to inflammation. There is evidence that IDO is increased in tumours and that a higher IDO expression has been associated with poorer prognosis [139,140]. The development of IDO inhibitors has therefore been investigated and clinical studies have begun [141]. Preliminary data suggest the IDO inhibitors indoximod and epacadostat to be safe and well tolerated, with some evidence of stable disease [142,143]. However, it is too early to speculate on the success of these drugs as standalone therapies and their influence on the anti-tumour immune responses. Combination of IDO inhibitors and checkpoint blockade are currently being pursued in the clinic.

## 5. Combination Therapies

The success of checkpoint blockade agents is unprecedented and has been accompanied by an exponential increase in the number of clinical trials using immunotherapies. The focus of these studies is increasingly moving towards combination therapies that utilise multiple checkpoint blockade therapies or combine checkpoint blockade with chemotherapy, radiotherapy, or immunotherapy. There are currently over 1000 clinical trials that combine PDL1/PD-1 therapies with other agents. The majority of these trials combine PD1/PDL1 therapy with CTLA-4 or chemotherapy therapy [144]. In preclinical trials combination of PD-1 and CTLA-4 therapies was shown to have a synergistic effect on B16 melanoma tumour growth [145]. The FDA has approved the combination of nivolumab and ipilimumab for treatment of melanoma based on the improved outcome when compared to patients treated with ipilimumab alone [146]. The combination of the anti-PD-1 drug Pembrolizumab and chemotherapy has recently been shown to be an effective and relatively well tolerated first line treatment for non-squamous non-small cell lung cancer (NSCLC) [147].

Checkpoint inhibitors rely upon the presence of an existing immune response that can be uncovered or modulated by the therapy. Therefore, they are likely to be more effective at treating immunologically “hot” tumours. In fact it has been suggested that the high occurrence of immune cold tumours may explain why checkpoint inhibitor studies have only shown a response rate of between 20–40% [148]. Overcoming the lack of tumour infiltrate in cold tumours is therefore necessary to increase the response rate. Combinations of checkpoint inhibitors are unlikely to address this issue. Recent evidence suggests that the solution may be to combine checkpoint inhibitors with treatments that are designed to induce an anti-tumour T-cell response, such as vaccines. The intention for these combination therapies is to generate a better immune response with the vaccine or other therapy and then to remove the suppressive effect of the tumour microenvironment with checkpoint inhibitors or other immune modulators.

Preclinical studies have demonstrated encouraging results for the combination of vaccines with checkpoint blockade [149,150,151,152]. In preclinical studies, checkpoint blockade has been shown to increase the efficacy of a number of different types of vaccine, including peptide and DNA based vaccines [153]. The GVAX vaccine that was used to target pancreatic ductal adenocarcinoma (PDA) was associated with increased PD-L1 expression in mice and combination with PD-1 blockade led to increased survival [154]. We have shown similar findings where a DNA vaccine promoted increased T-cell infiltration into tumours and increased PD-L1 expression by the tumour. The combination of vaccination with PD-1 blockade further enhanced the T-cell infiltrate and promoted better tumour therapy [151]. A similar phenomenon was seen with an alternative DNA vaccine in combination with PD-1 or CTLA-4 blockade [152]. Promising results have also been observed in the combination of checkpoint blockade with DC vaccine therapy and synthetic long peptide vaccination [155,156].

Combination of checkpoint inhibitors with other antigen specific therapies, including oncolytic viruses and CAR T-cells, are also being developed [157]. For oncolytic viruses, one phase 1b clinical trial has shown that combination of oncolytic viruses and anti-PD-1 treatment is well tolerated and increases the overall response rate [158]. In 21 patients with metastatic melanoma the response rate was 62% to a combination of T-vec and the anti-PD-1 antibody pembrolizumab. This has led to multiple phase 2 trials, which are in progress. For CAR T-cells blocking PD-1 has been shown to restore the CAR T-cell cytotoxicity in vitro [159].

Additional combination therapies include agents which reduce T-cell regulation by other methods. Concurrent with the clinical assessment of the IDO inhibitor indoximod as a standalone treatment it is also being assessed in combination with checkpoint blockade [160]. The combination of the IDO inhibitor epacadostat and PD-1 inhibitor pembrolizumab has shown promise in early clinical studies [161]. This combination has since advanced to phase III testing in the KEYNOTE-252/ECHO-301 trial (NCT02752074). The combination with nivolumab in the phase I/II ECHO-204 trial also showed promising early results in a number of solid tumors [162]. Findings presented at the American Society of Clinical Oncology in 2017 showed the combination demonstrated an objective response rate (ORR) of 63% and a clinical response (CR) rate of 5% among 74 patients with treatment-naïve melanoma. This combination is now being considered in phase III trial (NCT03301636). However, not all combination therapies include checkpoint inhibitors. One example is the combination of CAR T-cells with IDO therapies, which has shown that IDO expression can inhibit CAR T-cells and that administering fludarabine and cyclophosphamide can improve the efficacy of CAR T-cells by decreasing IDO expression [163].

Finally, the combination of immune agents with other treatments, including chemotherapy and radiotherapy, is being explored by a number of studies. Radiation in combination with immunotherapies has great potential as a combination therapy [148]. In mouse models of melanoma local radiation has been shown to increase antigen-specific T-cells against OVA-expressing B16 tumours [164]. Ablative radiotherapy has been shown to increase T-cell priming in draining lymph nodes, leading to an anti-tumour effect in mice [165]. Drugs like Temozolomide, which are used to treat cancer by impairing DNA mismatch repair (MMR), have been shown to lead to an increase in neo-antigens in the tumour, and therefore increased immune surveillance [166]. These studies lead to the obvious conclusion that chemotherapy drugs could work in combination with immunotherapies that exploit neo-antigens, such as checkpoint inhibitors [167].

All of these combination therapies require optimisation with some combination therapies proving effective where others have shown toxicity [127]. However, by combining therapies effectively and in well planned clinical trials it may be possible to accelerate delivery of effective therapies to patients [144].

## 6. Conclusions

In recent years the clinical success of checkpoint inhibitors has increased confidence in the efficacy of immunology based strategies for treating cancer. Despite the success with checkpoint inhibitors, many patients and tumour types have failed to show clinical responses. In fact, clinical trials using immunotherapies have highlighted the inherent challenges of using immune based techniques to enhance anti-cancer immunity. The key areas of research have focused on finding tumour specific targets and overcoming the anti-inflammatory tumour microenvironment, and both of these have proved challenging. However, in order to generate a robust immune response that is able to eliminate cancer and prevent regression, future clinical programs will likely involve combinations of these strategies. A better understanding of individual tumours and their T-cell infiltrate will be key in determining treatment options and the correct combination of treatments.

## Figures and Tables

**Table 1:** List of Approved Checkpoint Inhibitors.

Name	Target	Company	U.S. Food and Drug Administration (FDA) Approved Indications	E.U. Approved Indications
Pembrolizumab	PD-1	Merck (MSD)	Inoperable or metastatic melanoma; Metastatic non-small cell lung cancer with PDL-1 expression; Metastatic non-squamous non-small cell lung cancer; Metastatic non-small cell lung cancer with high PD-L1 expression; Recurrent or metastatic head and neck squamous cell carcinoma; Refractory classical Hodgkin lymphoma; Locally advanced or metastatic urothelial carcinoma; Microsatellite instability-high (MSI-H) or mismatch-repair deficient (dMMR) cancers; Recurrent locally advanced or metastatic gastric or gastroesophageal junction (GEJ) adenocarcinoma	Inoperable or metastatic melanoma; Metastatic non-squamous non-small cell lung cancer
Nivolumab	PD-1	Bristol-Myers Squibb	Inoperable of metastatic melanoma; Metastatic non-small cell lung cancer; Advanced renal cell carcinoma; Classical Hodgkin lymphoma; Recurrent or metastatic head and neck squamous cell carcinoma; Locally advanced or metastatic urothelial carcinoma; Microsatallite instability-high (MSI-H) or mismatch-repair deficient (dMMR) cancers; Hepatocarcinoma	Inoperable or metastatic melanoma; Metastatic non-small cell lung cancer; Advanced renal cell carcinoma; Classical Hodgkin lymphoma
Ipilimumab	CTLA-4	Bristol-Myers Squibb	Inoperable or metastatic melanoma	Inoperable or metastatic melanoma
Atezolizumab	PD-L1	Roche Genentech	Locally advanced or metastatic urothelial carcinoma; Metastatic non-small cell lung cancer	
Avelumab	PDL1	Merck Serono Pfizer	Metastatic Mercel cell carcinoma (MCC); Locally advanced or metastatic urothelial carcinoma	
Durvalumab	PD-L1	AstraZeneca	Locally advanced or metastatic urothelial carcinoma	

## References

[1] Ichim C.V. (2005). Revisiting immunosurveillance and immunostimulation: Implications for cancer immunotherapy. J. Trans. Med..

[2] Burnet F.M. (1970). The concept of immunological surveillance. Prog. Exp. Tumor. Res..

[3] Swann J.B., Smyth M.J. (2007). Immune surveillance of tumors. J. Clin. Investig..

[4] Shankaran V., Ikeda H., Bruce A.T., White J.M., Swanson P.E., Old L.J., Schreiber R.D. (2001). IFNgamma and lymphocytes prevent primary tumour development and shape tumour immunogenicity. Nature.

[5] Ferradini L., Mackensen A., Genevee C., Bosq J., Duvillard P., Avril M.F., Hercend T. (1993). Analysis of T cell receptor variability in tumor-infiltrating lymphocytes from a human regressive melanoma. Evidence for in situ T cell clonal expansion. J. Clin. Investig..

[6] Zorn E., Hercend T. (1999). A natural cytotoxic T cell response in a spontaneously regressing human melanoma targets a neoantigen resulting from a somatic point mutation. Eur. J. Immunol..

[7] Schreiber R.D., Old L.J., Smyth M.J. (2011). Cancer immunoediting: Integrating immunity’s roles in cancer suppression and promotion. Science.

[8] Alexandrov L.B., Nik-Zainal S., Wedge D.C., Aparicio S.A., Behjati S., Biankin A.V., Bignell G.R., Bolli N., Borg A., Borresen-Dale A.L. (2013). Signatures of mutational processes in human cancer. Nature.

[9] Rizvi N.A., Hellmann M.D., Snyder A., Kvistborg P., Makarov V., Havel J.J., Lee W., Yuan J., Wong P., Ho T.S. (2015). Cancer immunology. Mutational landscape determines sensitivity to PD-1 blockade in non-small cell lung cancer. Science.

[10] Siemers N.O., Holloway J.L., Chang H., Chasalow S.D., Ross-MacDonald P.B., Voliva C.F., Szustakowski J.D. (2017). Genome-wide association analysis identifies genetic correlates of immune infiltrates in solid tumors. PLoS ONE.

[11] Galon J., Lugli A., Bifulco C., Pages F., Masucci G., Marincola F.M., Ascierto P.A. (2017). World-Wide Immunoscore Task Force: Meeting report from the “Melanoma Bridge”, Napoli, November 30th-December 3rd, 2016. J. Trans. Med..

[12] Lanitis E., Dangaj D., Irving M., Coukos G. (2017). Mechanisms regulating T-cell infiltration and activity in solid tumors. Ann. Oncol.

[13] Ji R.R., Chasalow S.D., Wang L., Hamid O., Schmidt H., Cogswell J., Alaparthy S., Berman D., Jure-Kunkel M., Siemers N.O. (2012). An immune-active tumor microenvironment favors clinical response to ipilimumab. Cancer Immunol. Immunother..

[14] Driessens G., Kline J., Gajewski T.F. (2009). Costimulatory and coinhibitory receptors in anti-tumor immunity. Immunol. Rev..

[15] Capece D., Verzella D., Fischietti M., Zazzeroni F., Alesse E. (2012). Targeting costimulatory molecules to improve antitumor immunity. J. Biomed. Biotechnol..

[16] Shiao S.L., Chu G.C., Chung L.W. (2016). Regulation of prostate cancer progression by the tumor microenvironment. Cancer Lett..

[17] Taube J.M., Galon J., Sholl L.M., Rodig S.J., Cottrell T.R., Giraldo N.A., Baras A.S., Patel S.S., Anders R.A., Rimm D.L. (2017). Implications of the tumor immune microenvironment for staging and therapeutics. Mod. Pathol..

[18] Bahrami A., Khazaei M., Hassanian S.M., ShahidSales S., Joudi-Mashhad M., Maftouh M., Jazayeri M.H., Parizade M.R., Ferns G.A., Avan A. (2018). Targeting the tumor microenvironment as a potential therapeutic approach in colorectal cancer: Rational and progress. J. Cell. Physiol..

[19] Jarmalavicius S., Welte Y., Walden P. (2012). High immunogenicity of the human leukocyte antigen peptidomes of melanoma tumor cells. J. Biol. Chem..

[20] Lennerz V., Fatho M., Gentilini C., Frye R.A., Lifke A., Ferel D., Wolfel C., Huber C., Wolfel T. (2005). The response of autologous T cells to a human melanoma is dominated by mutated neoantigens. Proc. Natl. Acad. Sci. USA.

[21] Łuksza M.R.N., Makarov V., Balachandran V.P., Hellman H., Solovyov A., Rizvi N.A., Merghoub T., Levine A.J., Chan T.A., Greenbaum B.D. (2017). A neoantigen fitness model predicts tumor response to checkpoint blockade immunotherapy. Nature.

[22] Bassani-Sternberg M., Braunlein E., Klar R., Engleitner T., Sinitcyn P., Audehm S., Straub M., Weber J., Slotta-Huspenina J., Specht K. (2016). Direct identification of clinically relevant neoepitopes presented on native human melanoma tissue by mass spectrometry. Nat. Commun..

[23] Carreno B.M., Magrini V., Becker-Hapak M., Kaabinejadian S., Hundal J., Petti A.A., Ly A., Lie W.R., Hildebrand W.H., Mardis E.R. (2015). Cancer immunotherapy. A dendritic cell vaccine increases the breadth and diversity of melanoma neoantigen-specific T cells. Science.

[24] Sahin U., Derhovanessian E., Miller M., Kloke B.P., Simon P., Lower M., Bukur V., Tadmor A.D., Luxemburger U., Schrors B. (2017). Personalized RNA mutanome vaccines mobilize poly-specific therapeutic immunity against cancer. Nature.

[25] Ott P.A., Hu Z., Keskin D.B., Shukla S.A., Sun J., Bozym D.J., Zhang W., Luoma A., Giobbie-Hurder A., Peter L. (2017). An immunogenic personal neoantigen vaccine for patients with melanoma. Nature.

[26] Kreiter S., Vormehr M., van de Roemer N., Diken M., Lower M., Diekmann J., Boegel S., Schrors B., Vascotto F., Castle J.C. (2015). Mutant MHC class II epitopes drive therapeutic immune responses to cancer. Nature.

[27] Verdegaal E.M., de Miranda N.F., Visser M., Harryvan T., van Buuren M.M., Andersen R.S., Hadrup S.R., van der Minne C.E., Schotte R., Spits H. (2016). Neoantigen landscape dynamics during human melanoma-T cell interactions. Nature.

[28] Depontieu F.R., Qian J., Zarling A.L., McMiller T.L., Salay T.M., Norris A., English A.M., Shabanowitz J., Engelhard V.H., Hunt D.F. (2009). Identification of tumor-associated, MHC class II-restricted phosphopeptides as targets for immunotherapy. Proc. Natl. Acad. Sci. USA.

[29] Zarling A.L., Obeng R.C., Desch A.N., Pinczewski J., Cummings K.L., Deacon D.H., Conaway M., Slingluff C.L., Engelhard V.H. (2014). MHC-restricted phosphopeptides from insulin receptor substrate-2 and CDC25b offer broad-based immunotherapeutic agents for cancer. Cancer Res..

[30] Cobbold M., De La Pena H., Norris A., Polefrone J.M., Qian J., English A.M., Cummings K.L., Penny S., Turner J.E., Cottine J. (2013). MHC class I-associated phosphopeptides are the targets of memory-like immunity in leukemia. Sci. Trans. Med..

[31] Labots M., van der Mijn J.C., Beekhof R., Piersma S.R., de Goeij-de Haas R.R., Pham T.V., Knol J.C., Dekker H., van Grieken N.C.T., Verheul H.M.W. (2017). Phosphotyrosine-based-phosphoproteomics scaled-down to biopsy level for analysis of individual tumor biology and treatment selection. J. Proteomics.

[32] Witalison E.E., Thompson P.R., Hofseth L.J. (2015). Protein Arginine Deiminases and Associated Citrullination: Physiological Functions and Diseases Associated with Dysregulation. Curr. Drug Targets.

[33] Durrant L.G., Metheringham R.L., Brentville V.A. (2016). Autophagy, citrullination and cancer. Autophagy.

[34] Ireland J.M., Unanue E.R. (2011). Autophagy in antigen-presenting cells results in presentation of citrullinated peptides to CD4 T cells. J. Exp. Med..

[35] Brentville V.A., Metheringham R.L., Gunn B., Symonds P., Daniels I., Gijon M., Cook K., Xue W., Durrant L.G. (2016). Citrullinated Vimentin Presented on MHC-II in Tumor Cells Is a Target for CD4+ T-Cell-Mediated Antitumor Immunity. Cancer Res..

[36] Cook K., Daniels I., Symonds P., Pitt T., Gijon M., Xue W., Metheringham R., Durrant L., Brentville V. (2018). Citrullinated alpha-enolase is an effective target for anti-cancer immunity. Oncoimmunology.

[37] Goeb V., Thomas-L’Otellier M., Daveau R., Charlionet R., Fardellone P., Le Loet X., Tron F., Gilbert D., Vittecoq O. (2009). Candidate autoantigens identified by mass spectrometry in early rheumatoid arthritis are chaperones and citrullinated glycolytic enzymes. Arthritis Res. Ther..

[38] Rondas D., Crevecoeur I., D’Hertog W., Ferreira G.B., Staes A., Garg A.D., Eizirik D.L., Agostinis P., Gevaert K., Overbergh L. (2015). Citrullinated glucose-regulated protein 78 is an autoantigen in type 1 diabetes. Diabetes.

[39] Carter C.A., Oronsky B.T., Roswarski J., Oronsky A.L., Oronsky N., Scicinski J., Lybeck H., Kim M.M., Lybeck M., Reid T.R. (2017). No patient left behind: The promise of immune priming with epigenetic agents. Oncoimmunology.

[40] Malaker S.A., Penny S.A., Steadman L.G., Myers P.T., Loke J.C., Raghavan M., Bai D.L., Shabanowitz J., Hunt D.F., Cobbold M. (2017). Identification of Glycopeptides as Posttranslationally Modified Neoantigens in Leukemia. Cancer Immunol. Res..

[41] Boon T., van der Bruggen P. (1996). Human tumor antigens recognized by T lymphocytes. J. Exp. Med..

[42] Khong H.T., Rosenberg S.A. (2002). Pre-existing immunity to tyrosinase-related protein (TRP)-2, a new TRP-2 isoform, and the NY-ESO-1 melanoma antigen in a patient with a dramatic response to immunotherapy. J. Immunol..

[43] Gnjatic S., Altorki N.K., Tang D.N., Tu S.M., Kundra V., Ritter G., Old L.J., Logothetis C.J., Sharma P. (2009). NY-ESO-1 DNA vaccine induces T-cell responses that are suppressed by regulatory T cells. Clin. Cancer Res..

[44] Jiang J., Xie D., Zhang W., Xiao G., Wen J. (2013). Fusion of Hsp70 to Mage-a1 enhances the potency of vaccine-specific immune responses. J. Trans. Med..

[45] Patel P.M., Ottensmeier C.H., Mulatero C., Lorigan P., Plummer R., Pandha H., Elsheikh S., Hadjimichael E., Villasanti N., Adams S.E. (2018). Targeting gp100 and TRP-2 with a DNA vaccine: Incorporating T cell epitopes with a human IgG1 antibody induces potent T cell responses that are associated with favourable clinical outcome in a phase I/II trial. Oncoimmunology.

[46] Nishimura Y., Tomita Y., Yuno A., Yoshitake Y., Shinohara M. (2015). Cancer immunotherapy using novel tumor-associated antigenic peptides identified by genome-wide cDNA microarray analyses. Cancer Sci..

[47] Yoshitake Y., Fukuma D., Yuno A., Hirayama M., Nakayama H., Tanaka T., Nagata M., Takamune Y., Kawahara K., Nakagawa Y. (2015). Phase II clinical trial of multiple peptide vaccination for advanced head and neck cancer patients revealed induction of immune responses and improved OS. Clin. Cancer Res..

[48] Yoshitake Y., Nishimura Y., Nakamura Y., Shinohara M. (2015). A clinical trial of multiple peptides vaccination for advanced head and neck cancer patients induced immune responses and prolonged OS. Oncoimmunology.

[49] White J.R., Winter J.A., Robinson K. (2015). Differential inflammatory response to Helicobacter pylori infection: Etiology and clinical outcomes. J. Inflamm. Res..

[50] Schiller J.T., Castellsague X., Garland S.M. (2012). A review of clinical trials of human papillomavirus prophylactic vaccines. Vaccine.

[51] Van Poelgeest M.I., Welters M.J., van Esch E.M., Stynenbosch L.F., Kerpershoek G., van Persijn van Meerten E.L., van den Hende M., Lowik M.J., Berends-van der Meer D.M., Fathers L.M. (2013). HPV16 synthetic long peptide (HPV16-SLP) vaccination therapy of patients with advanced or recurrent HPV16-induced gynecological carcinoma, a phase II trial. J. Trans. Med..

[52] Chia W.K., Wang W.W., Teo M., Tai W.M., Lim W.T., Tan E.H., Leong S.S., Sun L., Chen J.J., Gottschalk S. (2012). A phase II study evaluating the safety and efficacy of an adenovirus-DeltaLMP1-LMP2 transduced dendritic cell vaccine in patients with advanced metastatic nasopharyngeal carcinoma. Ann. Oncol..

[53] Taylor G.S., Jia H., Harrington K., Lee L.W., Turner J., Ladell K., Price D.A., Tanday M., Matthews J., Roberts C. (2014). A recombinant modified vaccinia ankara vaccine encoding Epstein-Barr Virus (EBV) target antigens: A phase I trial in UK patients with EBV-positive cancer. Clin. Cancer Res..

[54] Smola S., Trimble C., Stern P.L. (2017). Human papillomavirus-driven immune deviation: Challenge and novel opportunity for immunotherapy. Ther. Adv. Vaccines.

[55] Tashiro H., Brenner M.K. (2017). Immunotherapy against cancer-related viruses. Cell Res..

[56] Dowling J.K., Mansell A. (2016). Toll-like receptors: The swiss army knife of immunity and vaccine development. Clin. Trans. Immunol..

[57] Zom G.G., Filippov D.V., van der Marel G.A., Overkleeft H.S., Melief C.J., Ossendorp F. (2014). Two in one: Improving synthetic long peptide vaccines by combining antigen and adjuvant in one molecule. Oncoimmunology.

[58] Zom G.G., Welters M.J., Loof N.M., Goedemans R., Lougheed S., Valentijn R.R., Zandvliet M.L., Meeuwenoord N.J., Melief C.J., de Gruijl T.D. (2016). TLR2 ligand-synthetic long peptide conjugates effectively stimulate tumor-draining lymph node T cells of cervical cancer patients. Oncotarget.

[59] Cho H.I., Barrios K., Lee Y.R., Linowski A.K., Celis E. (2013). BiVax: A peptide/poly-IC subunit vaccine that mimics an acute infection elicits vast and effective anti-tumor CD8 T-cell responses. Cancer Immunol. Immunother..

[60] Kumai T., Lee S., Cho H.I., Sultan H., Kobayashi H., Harabuchi Y., Celis E. (2017). Optimization of Peptide Vaccines to Induce Robust Antitumor CD4 T-cell Responses. Cancer Immunol. Res..

[61] Pudney V.A., Metheringham R.L., Gunn B., Spendlove I., Ramage J.M., Durrant L.G. (2010). DNA vaccination with T-cell epitopes encoded within Ab molecules induces high-avidity anti-tumor CD8+ T cells. Eur. J. Immunol..

[62] Bijker M.S., van den Eeden S.J., Franken K.L., Melief C.J., Offringa R., van der Burg S.H. (2007). CD8+ CTL priming by exact peptide epitopes in incomplete Freund’s adjuvant induces a vanishing CTL response, whereas long peptides induce sustained CTL reactivity. J. Immunol..

[63] Sabbatini P., Tsuji T., Ferran L., Ritter E., Sedrak C., Tuballes K., Jungbluth A.A., Ritter G., Aghajanian C., Bell-McGuinn K. (2012). Phase I trial of overlapping long peptides from a tumor self-antigen and poly-ICLC shows rapid induction of integrated immune response in ovarian cancer patients. Clin. Cancer Res..

[64] Tsuji T., Sabbatini P., Jungbluth A.A., Ritter E., Pan L., Ritter G., Ferran L., Spriggs D., Salazar A.M., Gnjatic S. (2013). Effect of Montanide and poly-ICLC adjuvant on human self/tumor antigen-specific CD4+ T cells in phase I overlapping long peptide vaccine trial. Cancer Immunol. Res..

[65] Rahma O.E., Hamilton J.M., Wojtowicz M., Dakheel O., Bernstein S., Liewehr D.J., Steinberg S.M., Khleif S.N. (2014). The immunological and clinical effects of mutated ras peptide vaccine in combination with IL-2, GM-CSF, or both in patients with solid tumors. J. Trans. Med..

[66] Schuster J., Lai R.K., Recht L.D., Reardon D.A., Paleologos N.A., Groves M.D., Mrugala M.M., Jensen R., Baehring J.M., Sloan A. (2015). A phase II, multicenter trial of rindopepimut (CDX-110) in newly diagnosed glioblastoma: The ACT III study. Neuro Oncol..

[67] Shang N., Figini M., Shangguan J., Wang B., Sun C., Pan L., Ma Q., Zhang Z. (2017). Dendritic cells based immunotherapy. Am. J. Cancer Res..

[68] Handy C.E., Antonarakis E.S. (2017). Sipuleucel-T for the treatment of prostate cancer: Novel insights and future directions. Future Oncol..

[69] Wimmers F., Schreibelt G., Skold A.E., Figdor C.G., De Vries I.J. (2014). Paradigm Shift in Dendritic Cell-Based Immunotherapy: From in vitro Generated Monocyte-Derived DCs to Naturally Circulating DC Subsets. Front. Immunol..

[70] Roos A.K., Eriksson F., Timmons J.A., Gerhardt J., Nyman U., Gudmundsdotter L., Brave A., Wahren B., Pisa P. (2009). Skin electroporation: Effects on transgene expression, DNA persistence and local tissue environment. PLoS ONE.

[71] Cany J., Barteau B., Tran L., Gauttier V., Archambeaud I., Couty J.P., Turlin B., Pitard B., Vassaux G., Ferry N. (2011). AFP-specific immunotherapy impairs growth of autochthonous hepatocellular carcinoma in mice. J. Hepatol..

[72] Pitard B. (2002). Supramolecular assemblies of DNA delivery systems. Somat. Cell Mol. Genet..

[73] Metheringham R.L., Pudney V.A., Gunn B., Towey M., Spendlove I., Durrant L.G. (2009). Antibodies designed as effective cancer vaccines. MAbs.

[74] Chudley L., McCann K., Mander A., Tjelle T., Campos-Perez J., Godeseth R., Creak A., Dobbyn J., Johnson B., Bass P. (2012). DNA fusion-gene vaccination in patients with prostate cancer induces high-frequency CD8(+) T-cell responses and increases PSA doubling time. Cancer Immunol. Immunother..

[75] McCann K.J., Mander A., Cazaly A., Chudley L., Stasakova J., Thirdborough S., King A., Lloyd-Evans P., Buxton E., Edwards C. (2016). Targeting Carcinoembryonic Antigen with DNA Vaccination: On-Target Adverse Events Link with Immunologic and Clinical Outcomes. Clin. Cancer Res..

[76] Hess P.R., Boczkowski D., Nair S.K., Snyder D., Gilboa E. (2006). Vaccination with mRNAs encoding tumor-associated antigens and granulocyte-macrophage colony-stimulating factor efficiently primes CTL responses, but is insufficient to overcome tolerance to a model tumor/self antigen. Cancer Immunol. Immunother..

[77] Fukuhara H., Ino Y., Todo T. (2016). Oncolytic virus therapy: A new era of cancer treatment at dawn. Cancer Sci..

[78] Liu B.L., Robinson M., Han Z.Q., Branston R.H., English C., Reay P., McGrath Y., Thomas S.K., Thornton M., Bullock P. (2003). ICP34.5 deleted herpes simplex virus with enhanced oncolytic, immune stimulating, and anti-tumour properties. Gen. Ther..

[79] Kaufman H.L., Bines S.D. (2010). OPTIM trial: A Phase III trial of an oncolytic herpes virus encoding GM-CSF for unresectable stage III or IV melanoma. Future Oncol..

[80] Andtbacka R.H., Kaufman H.L., Collichio F., Amatruda T., Senzer N., Chesney J., Delman K.A., Spitler L.E., Puzanov I., Agarwala S.S. (2015). Talimogene Laherparepvec Improves Durable Response Rate in Patients With Advanced Melanoma. J. Clin. Oncol..

[81] Foloppe J., Kintz J., Futin N., Findeli A., Cordier P., Schlesinger Y., Hoffmann C., Tosch C., Balloul J.M., Erbs P. (2008). Targeted delivery of a suicide gene to human colorectal tumors by a conditionally replicating vaccinia virus. Gen. Ther..

[82] Heinrich B., Klein J., Delic M., Goepfert K., Engel V., Geberzahn L., Lusky M., Erbs P., Preville X., Moehler M. (2017). Immunogenicity of oncolytic vaccinia viruses JX-GFP and TG6002 in a human melanoma in vitro model: Studying immunogenic cell death, dendritic cell maturation and interaction with cytotoxic T lymphocytes. Onco Target. Ther..

[83] Kirn D.H., Thorne S.H. (2009). Targeted and armed oncolytic poxviruses: A novel multi-mechanistic therapeutic class for cancer. Nat. Rev. Cancer.

[84] Kim J.H., Oh J.Y., Park B.H., Lee D.E., Kim J.S., Park H.E., Roh M.S., Je J.E., Yoon J.H., Thorne S.H. (2006). Systemic armed oncolytic and immunologic therapy for cancer with JX-594, a targeted poxvirus expressing GM-CSF. Mol. Ther..

[85] Heo J., Reid T., Ruo L., Breitbach C.J., Rose S., Bloomston M., Cho M., Lim H.Y., Chung H.C., Kim C.W. (2013). Randomized dose-finding clinical trial of oncolytic immunotherapeutic vaccinia JX-594 in liver cancer. Nat. Med..

[86] Gong J., Sachdev E., Mita A.C., Mita M.M. (2016). Clinical development of reovirus for cancer therapy: An oncolytic virus with immune-mediated antitumor activity. World J. Methodol..

[87] Karapanagiotou E.M., Roulstone V., Twigger K., Ball M., Tanay M., Nutting C., Newbold K., Gore M.E., Larkin J., Syrigos K.N. (2012). Phase I/II trial of carboplatin and paclitaxel chemotherapy in combination with intravenous oncolytic reovirus in patients with advanced malignancies. Clin. Cancer Res..

[88] Taguchi S., Fukuhara H., Homma Y., Todo T. (2017). Current status of clinical trials assessing oncolytic virus therapy for urological cancers. Int. J. Urol..

[89] Russell S.J., Federspiel M.J., Peng K.W., Tong C., Dingli D., Morice W.G., Lowe V., O’Connor M.K., Kyle R.A., Leung N. (2014). Remission of disseminated cancer after systemic oncolytic virotherapy. Mayo Clin. Proc..

[90] Hirasawa K., Nishikawa S.G., Norman K.L., Coffey M.C., Thompson B.G., Yoon C.S., Waisman D.M., Lee P.W. (2003). Systemic reovirus therapy of metastatic cancer in immune-competent mice. Cancer Res..

[91] Hinrichs C.S., Rosenberg S.A. (2014). Exploiting the curative potential of adoptive T-cell therapy for cancer. Immunol. Rev..

[92] Chandran S.S., Paria B.C., Srivastava A.K., Rothermel L.D., Stephens D.J., Dudley M.E., Somerville R., Wunderlich J.R., Sherry R.M., Yang J.C. (2015). Persistence of CTL clones targeting melanocyte differentiation antigens was insufficient to mediate significant melanoma regression in humans. Clin. Cancer Res..

[93] Robbins P.F., Lu Y.C., El-Gamil M., Li Y.F., Gross C., Gartner J., Lin J.C., Teer J.K., Cliften P., Tycksen E. (2013). Mining exomic sequencing data to identify mutated antigens recognized by adoptively transferred tumor-reactive T cells. Nat. Med..

[94] Prickett T.D., Crystal J.S., Cohen C.J., Pasetto A., Parkhurst M.R., Gartner J.J., Yao X., Wang R., Gros A., Li Y.F. (2016). Durable Complete Response from Metastatic Melanoma after Transfer of Autologous T Cells Recognizing 10 Mutated Tumor Antigens. Cancer Immunol. Res..

[95] Johnson L.A., Morgan R.A., Dudley M.E., Cassard L., Yang J.C., Hughes M.S., Kammula U.S., Royal R.E., Sherry R.M., Wunderlich J.R. (2009). Gene therapy with human and mouse T-cell receptors mediates cancer regression and targets normal tissues expressing cognate antigen. Blood.

[96] Morgan R.A., Dudley M.E., Wunderlich J.R., Hughes M.S., Yang J.C., Sherry R.M., Royal R.E., Topalian S.L., Kammula U.S., Restifo N.P. (2006). Cancer regression in patients after transfer of genetically engineered lymphocytes. Science.

[97] Raman M.C., Rizkallah P.J., Simmons R., Donnellan Z., Dukes J., Bossi G., Le Provost G.S., Todorov P., Baston E., Hickman E. (2016). Direct molecular mimicry enables off-target cardiovascular toxicity by an enhanced affinity TCR designed for cancer immunotherapy. Sci. Rep..

[98] Van den Berg J.H., Gomez-Eerland R., van de Wiel B., Hulshoff L., van den Broek D., Bins A., Tan H.L., Harper J.V., Hassan N.J., Jakobsen B.K. (2015). Case Report of a Fatal Serious Adverse Event Upon Administration of T Cells Transduced With a MART-1-specific T-cell Receptor. Mol. Ther..

[99] Robbins P.F., Morgan R.A., Feldman S.A., Yang J.C., Sherry R.M., Dudley M.E., Wunderlich J.R., Nahvi A.V., Helman L.J., Mackall C.L. (2011). Tumor regression in patients with metastatic synovial cell sarcoma and melanoma using genetically engineered lymphocytes reactive with NY-ESO-1. J. Clin. Oncol..

[100] Zhang H., Snyder K.M., Suhoski M.M., Maus M.V., Kapoor V., June C.H., Mackall C.L. (2007). 4-1BB is superior to CD28 costimulation for generating CD8+ cytotoxic lymphocytes for adoptive immunotherapy. J. Immunol..

[101] Srivastava S., Riddell S.R. (2018). Chimeric Antigen Receptor T Cell Therapy: Challenges to Bench-to-Bedside Efficacy. J. Immunol..

[102] Park S., Shevlin E., Vedvyas Y., Zaman M., Park S., Hsu Y.S., Min I.M., Jin M.M. (2017). Micromolar affinity CAR T cells to ICAM-1 achieves rapid tumor elimination while avoiding systemic toxicity. Sci. Rep..

[103] Ritchie D.S., Neeson P.J., Khot A., Peinert S., Tai T., Tainton K., Chen K., Shin M., Wall D.M., Honemann D. (2013). Persistence and efficacy of second generation CAR T cell against the LeY antigen in acute myeloid leukemia. Mol. Ther..

[104] Grupp S.A., Kalos M., Barrett D., Aplenc R., Porter D.L., Rheingold S.R., Teachey D.T., Chew A., Hauck B., Wright J.F. (2013). Chimeric antigen receptor-modified T cells for acute lymphoid leukemia. N. Eng. J. Med..

[105] Pan J., Yang J.F., Deng B.P., Zhao X.J., Zhang X., Lin Y.H., Wu Y.N., Deng Z.L., Zhang Y.L., Liu S.H. (2017). High efficacy and safety of low-dose CD19-directed CAR-T cell therapy in 51 refractory or relapsed B acute lymphoblastic leukemia patients. Leukemia.

[106] Maude S.L., Laetsch T.W., Buechner J., Rives S., Boyer M., Bittencourt H., Bader P., Verneris M.R., Stefanski H.E., Myers G.D. (2018). Tisagenlecleucel in Children and Young Adults with B-Cell Lymphoblastic Leukemia. N. Eng. J. Med..

[107] Neelapu S.S., Locke F.L., Bartlett N.L., Lekakis L.J., Miklos D.B., Jacobson C.A., Braunschweig I., Oluwole O.O., Siddiqi T., Lin Y. (2017). Axicabtagene Ciloleucel CAR T-Cell Therapy in Refractory Large B-Cell Lymphoma. N. Eng. J. Med..

[108] Fan M., Li M., Gao L., Geng S., Wang J., Wang Y., Yan Z., Yu L. (2017). Chimeric antigen receptors for adoptive T cell therapy in acute myeloid leukemia. J. Hematol. Oncol..

[109] Mirzaei H.R., Rodriguez A., Shepphird J., Brown C.E., Badie B. (2017). Chimeric Antigen Receptors T Cell Therapy in Solid Tumor: Challenges and Clinical Applications. Front. Immunol..

[110] Yu J., Wu X., Yan J., Yu H., Xu L., Chi Z., Sheng X., Si L., Cui C., Dai J. (2018). Anti-GD2/4-1BB chimeric antigen receptor T cell therapy for the treatment of Chinese melanoma patients. J. Hematol. Oncol..

[111] Brown C.E., Alizadeh D., Starr R., Weng L., Wagner J.R., Naranjo A., Ostberg J.R., Blanchard M.S., Kilpatrick J., Simpson J. (2016). Regression of Glioblastoma after Chimeric Antigen Receptor T-Cell Therapy. N. Eng. J. Med..

[112] Brown C.E., Aguilar B., Starr R., Yang X., Chang W.C., Weng L., Chang B., Sarkissian A., Brito A., Sanchez J.F. (2018). Optimization of IL13Ralpha2-Targeted Chimeric Antigen Receptor T Cells for Improved Anti-tumor Efficacy against Glioblastoma. Mol. Ther..

[113] Irving M., Vuillefroy de Silly R., Scholten K., Dilek N., Coukos G. (2017). Engineering Chimeric Antigen Receptor T-Cells for Racing in Solid Tumors: Don’t Forget the Fuel. Front. Immunol..

[114] Lee P.P., Yee C., Savage P.A., Fong L., Brockstedt D., Weber J.S., Johnson D., Swetter S., Thompson J., Greenberg P.D. (1999). Characterization of circulating T cells specific for tumor-associated antigens in melanoma patients. Nat. Med..

[115] Linch S.N., McNamara M.J., Redmond W.L. (2015). OX40 Agonists and Combination Immunotherapy: Putting the Pedal to the Metal. Front. Oncol..

[116] Weinberg A.D., Rivera M.M., Prell R., Morris A., Ramstad T., Vetto J.T., Urba W.J., Alvord G., Bunce C., Shields J. (2000). Engagement of the OX-40 receptor in vivo enhances antitumor immunity. J. Immunol..

[117] Thomas L.J., He L.Z., Marsh H., Keler T. (2014). Targeting human CD27 with an agonist antibody stimulates T-cell activation and antitumor immunity. Oncoimmunology.

[118] Buchan S.L., Rogel A., Al-Shamkhani A. (2018). The immunobiology of CD27 and OX40 and their potential as targets for cancer immunotherapy. Blood.

[119] Chester C., Sanmamed M.F., Wang J., Melero I. (2018). Immunotherapy targeting 4-1BB: Mechanistic rationale, clinical results, and future strategies. Blood.

[120] Milling L., Zhang Y., Irvine D.J. (2017). Delivering safer immunotherapies for cancer. Adv. Drug Deliv. Rev..

[121] Kvistborg P., Philips D., Kelderman S., Hageman L., Ottensmeier C., Joseph-Pietras D., Welters M.J., van der Burg S., Kapiteijn E., Michielin O. (2014). Anti-CTLA-4 therapy broadens the melanoma-reactive CD8+ T cell response. Sci. Trans. Med..

[122] Simpson T.R., Li F., Montalvo-Ortiz W., Sepulveda M.A., Bergerhoff K., Arce F., Roddie C., Henry J.Y., Yagita H., Wolchok J.D. (2013). Fc-dependent depletion of tumor-infiltrating regulatory T cells co-defines the efficacy of anti-CTLA-4 therapy against melanoma. J. Exp. Med..

[123] Sharma P., Allison J.P. (2015). The future of immune checkpoint therapy. Science.

[124] Yoest J.M. (2017). Clinical features, predictive correlates, and pathophysiology of immune-related adverse events in immune checkpoint inhibitor treatments in cancer: A short review. Immunotarget. Ther..

[125] Patsoukis N., Brown J., Petkova V., Liu F., Li L., Boussiotis V.A. (2012). Selective effects of PD-1 on Akt and Ras pathways regulate molecular components of the cell cycle and inhibit T cell proliferation. Sci. Signal..

[126] Chen L., Han X. (2015). Anti-PD-1/PD-L1 therapy of human cancer: Past, present, and future. J. Clin. Investig..

[127] Brown J.S., Sundar R., Lopez J. (2017). Combining DNA damaging therapeutics with immunotherapy: More haste, less speed. Br. J. Cancer.

[128] Hodi F.S., O’Day S.J., McDermott D.F., Weber R.W., Sosman J.A., Haanen J.B., Gonzalez R., Robert C., Schadendorf D., Hassel J.C. (2010). Improved survival with ipilimumab in patients with metastatic melanoma. N. Eng. J. Med..

[129] Robert C., Thomas L., Bondarenko I., O’Day S., Weber J., Garbe C., Lebbe C., Baurain J.F., Testori A., Grob J.J. (2011). Ipilimumab plus dacarbazine for previously untreated metastatic melanoma. N. Eng. J. Med..

[130] Takeuchi Y., Nishikawa H. (2016). Roles of regulatory T cells in cancer immunity. Int. Immunol..

[131] Shimizu J., Yamazaki S., Sakaguchi S. (1999). Induction of tumor immunity by removing CD25+CD4+ T cells: A common basis between tumor immunity and autoimmunity. J. Immunol..

[132] Peggs K.S., Quezada S.A., Chambers C.A., Korman A.J., Allison J.P. (2009). Blockade of CTLA-4 on both effector and regulatory T cell compartments contributes to the antitumor activity of anti-CTLA-4 antibodies. J. Exp. Med..

[133] Parker K.H., Beury D.W., Ostrand-Rosenberg S. (2015). Myeloid-Derived Suppressor Cells: Critical Cells Driving Immune Suppression in the Tumor Microenvironment. Adv. Cancer Res..

[134] Annels N.E., Shaw V.E., Gabitass R.F., Billingham L., Corrie P., Eatock M., Valle J., Smith D., Wadsley J., Cunningham D. (2014). The effects of gemcitabine and capecitabine combination chemotherapy and of low-dose adjuvant GM-CSF on the levels of myeloid-derived suppressor cells in patients with advanced pancreatic cancer. Cancer Immunol. Immunother..

[135] Iclozan C., Antonia S., Chiappori A., Chen D.T., Gabrilovich D. (2013). Therapeutic regulation of myeloid-derived suppressor cells and immune response to cancer vaccine in patients with extensive stage small cell lung cancer. Cancer Immunol. Immunother..

[136] Weed D.T., Vella J.L., Reis I.M., De la Fuente A.C., Gomez C., Sargi Z., Nazarian R., Califano J., Borrello I., Serafini P. (2015). Tadalafil reduces myeloid-derived suppressor cells and regulatory T cells and promotes tumor immunity in patients with head and neck squamous cell carcinoma. Clin. Cancer Res..

[137] Schilling B., Sucker A., Griewank K., Zhao F., Weide B., Gorgens A., Giebel B., Schadendorf D., Paschen A. (2013). Vemurafenib reverses immunosuppression by myeloid derived suppressor cells. Int. J. Cancer.

[138] Munn D.H., Mellor A.L. (2016). IDO in the Tumor Microenvironment: Inflammation, Counter-Regulation, and Tolerance. Trends Immunol..

[139] Ino K., Yoshida N., Kajiyama H., Shibata K., Yamamoto E., Kidokoro K., Takahashi N., Terauchi M., Nawa A., Nomura S. (2006). Indoleamine 2,3-dioxygenase is a novel prognostic indicator for endometrial cancer. Br. J. Cancer.

[140] Liu H., Shen Z., Wang Z., Wang X., Zhang H., Qin J., Qin X., Xu J., Sun Y. (2016). Increased expression of IDO associates with poor postoperative clinical outcome of patients with gastric adenocarcinoma. Sci. Rep..

[141] Vacchelli E., Aranda F., Eggermont A., Sautes-Fridman C., Tartour E., Kennedy E.P., Platten M., Zitvogel L., Kroemer G., Galluzzi L. (2014). Trial watch: IDO inhibitors in cancer therapy. Oncoimmunology.

[142] Beatty G.L., O’Dwyer P.J., Clark J., Shi J.G., Bowman K.J., Scherle P.A., Newton R.C., Schaub R., Maleski J., Leopold L. (2017). First-in-Human Phase I Study of the Oral Inhibitor of Indoleamine 2,3-Dioxygenase-1 Epacadostat (INCB024360) in Patients with Advanced Solid Malignancies. Clin. Cancer Res..

[143] Soliman H.H., Minton S.E., Han H.S., Ismail-Khan R., Neuger A., Khambati F., Noyes D., Lush R., Chiappori A.A., Roberts J.D. (2016). A phase I study of indoximod in patients with advanced malignancies. Oncotarget.

[144] Tang J., Shalabi A., Hubbard-Lucey V.M. (2018). Comprehensive analysis of the clinical immuno-oncology landscape. Ann. Oncol..

[145] Curran M.A., Montalvo W., Yagita H., Allison J.P. (2010). PD-1 and CTLA-4 combination blockade expands infiltrating T cells and reduces regulatory T and myeloid cells within B16 melanoma tumors. Proc. Nat. Acad. Sci. USA.

[146] Hodi F.S., Chesney J., Pavlick A.C., Robert C., Grossmann K.F., McDermott D.F., Linette G.P., Meyer N., Giguere J.K., Agarwala S.S. (2016). Combined nivolumab and ipilimumab versus ipilimumab alone in patients with advanced melanoma: 2-year overall survival outcomes in a multicentre, randomised, controlled, phase 2 trial. Lancet Oncol..

[147] Langer C.J., Gadgeel S.M., Borghaei H., Papadimitrakopoulou V.A., Patnaik A., Powell S.F., Gentzler R.D., Martins R.G., Stevenson J.P., Jalal S.I. (2016). Carboplatin and pemetrexed with or without pembrolizumab for advanced, non-squamous non-small-cell lung cancer: A randomised, phase 2 cohort of the open-label KEYNOTE-021 study. Lancet Oncol.

[148] Demaria S., Coleman C.N., Formenti S.C. (2016). Radiotherapy: Changing the Game in Immunotherapy. Trends Cancer.

[149] Binder D.C., Engels B., Arina A., Yu P., Slauch J.M., Fu Y.X., Karrison T., Burnette B., Idel C., Zhao M. (2013). Antigen-specific bacterial vaccine combined with anti-PD-L1 rescues dysfunctional endogenous T cells to reject long-established cancer. Cancer Immunol. Res..

[150] Duraiswamy J., Kaluza K.M., Freeman G.J., Coukos G. (2013). Dual blockade of PD-1 and CTLA-4 combined with tumor vaccine effectively restores T-cell rejection function in tumors. Cancer Res..

[151] Xue W., Brentville V.A., Symonds P., Cook K.W., Yagita H., Metheringham R.L., Durrant L.G. (2016). SCIB1, a huIgG1 antibody DNA vaccination, combined with PD-1 blockade induced efficient therapy of poorly immunogenic tumors. Oncotarget.

[152] Xue W., Metheringham R.L., Brentville V.A., Gunn B., Symonds P., Yagita H., Ramage J.M., Durrant L.G. (2016). SCIB2, an antibody DNA vaccine encoding NY-ESO-1 epitopes, induces potent antitumor immunity which is further enhanced by checkpoint blockade. Oncoimmunology.

[153] Karaki S., Anson M., Tran T., Giusti D., Blanc C., Oudard S., Tartour E. (2016). Is There Still Room for Cancer Vaccines at the Era of Checkpoint Inhibitors. Vaccines (Basel).

[154] Soares K.C., Rucki A.A., Wu A.A., Olino K., Xiao Q., Chai Y., Wamwea A., Bigelow E., Lutz E., Liu L. (2015). PD-1/PD-L1 blockade together with vaccine therapy facilitates effector T-cell infiltration into pancreatic tumors. J. Immunother..

[155] Ferris R.L., Blumenschein G., Fayette J., Guigay J., Colevas A.D., Licitra L., Harrington K., Kasper S., Vokes E.E., Even C. (2016). Nivolumab for Recurrent Squamous-Cell Carcinoma of the Head and Neck. N. Eng. J. Med..

[156] Wilgenhof S., Corthals J., Heirman C., van Baren N., Lucas S., Kvistborg P., Thielemans K., Neyns B. (2016). Phase II Study of Autologous Monocyte-Derived mRNA Electroporated Dendritic Cells (TriMixDC-MEL) Plus Ipilimumab in Patients With Pretreated Advanced Melanoma. J. Clin. Oncol..

[157] Chesney J., Puzanov I., Collichio F., Singh P., Milhem M.M., Glaspy J., Hamid O., Ross M., Friedlander P., Garbe C. (2017). Randomized, Open-Label Phase II Study Evaluating the Efficacy and Safety of Talimogene Laherparepvec in Combination With Ipilimumab Versus Ipilimumab Alone in Patients With Advanced, Unresectable Melanoma. J. Clin. Oncol..

[158] Ribas A., Dummer R., Puzanov I., VanderWalde A., Andtbacka R.H.I., Michielin O., Olszanski A.J., Malvehy J., Cebon J., Fernandez E. (2017). Oncolytic Virotherapy Promotes Intratumoral T Cell Infiltration and Improves Anti-PD-1 Immunotherapy. Cell.

[159] Moon E.K., Wang L.C., Dolfi D.V., Wilson C.B., Ranganathan R., Sun J., Kapoor V., Scholler J., Pure E., Milone M.C. (2014). Multifactorial T-cell hypofunction that is reversible can limit the efficacy of chimeric antigen receptor-transduced human T cells in solid tumors. Clin. Cancer Res..

[160] Davar D., Bahary N. (2018). Modulating Tumor Immunology by Inhibiting Indoleamine 2,3-Dioxygenase (IDO): Recent Developments and First Clinical Experiences. Target. Oncol..

[161] Gangadhar T.C.H., Smith D.C., Bauer T.M., Waser J.S., Luke J.J., Balmanoukian A.S., Kaufman D.R., Zhao Y., Maleski J., Leopold L. (2015). Preliminary results from a phase I/II study of epacadostat (INCB024360) in combination with pembrolizumab in patients with selected advanced cancers. J. Immunother. Cancer.

[162] Perez R.P., Riese M.J., Lewis K.D., Saleh M.N., Daud A., Berlin J., Lee J.J., Mukhopadhyay S., Zhou L., Serbest G. (2017). Epacadostat plus nivolumab in patients with advanced solid tumors: Preliminary phase I/II results of ECHO-204. J. Clin. Oncol..

[163] Ninomiya S., Narala N., Huye L., Yagyu S., Savoldo B., Dotti G., Heslop H.E., Brenner M.K., Rooney C.M., Ramos C.A. (2015). Tumor indoleamine 2,3-dioxygenase (IDO) inhibits CD19-CAR T cells and is downregulated by lymphodepleting drugs. Blood.

[164] Lugade A.A., Moran J.P., Gerber S.A., Rose R.C., Frelinger J.G., Lord E.M. (2005). Local radiation therapy of B16 melanoma tumors increases the generation of tumor antigen-specific effector cells that traffic to the tumor. J. Immunol..

[165] Lee Y., Auh S.L., Wang Y., Burnette B., Wang Y., Meng Y., Beckett M., Sharma R., Chin R., Tu T. (2009). Therapeutic effects of ablative radiation on local tumor require CD8+ T cells: Changing strategies for cancer treatment. Blood.

[166] Germano G., Lamba S., Rospo G., Barault L., Magri A., Maione F., Russo M., Crisafulli G., Bartolini A., Lerda G. (2017). Inactivation of DNA repair triggers neoantigen generation and impairs tumour growth. Nature.

[167] Franzese O., Torino F., Fuggetta M.P., Aquino A., Roselli M., Bonmassar E., Giuliani A., D’Atri S. (2017). Tumor immunotherapy: Drug-induced neoantigens (xenogenization) and immune checkpoint inhibitors. Oncotarget.

